# Design of a Rapid and Accurate Calibration System for Pressure Sensors with Minimized Temperature Variation

**DOI:** 10.3390/s25175288

**Published:** 2025-08-25

**Authors:** Juntong Cui, Shubin Zhang, Yanfeng Jiang

**Affiliations:** School of Integrated Circuits, Jiangnan University, Wuxi 214122, China; 1038220426@stu.jiangnan.edu.cn (J.C.); jiangyf@jiangnan.edu.cn (Y.J.)

**Keywords:** sensor conditioning, sensor calibration system, nonlinearity and zero drift, finite element method (FEM) simulation, temperature compensation

## Abstract

Miniaturized pressure sensors fabricated via micro-electro-mechanical systems (MEMSs) technology are ubiquitous in modern applications. However, the massively produced MEMS pressure sensors, prior to being practically used, need to be calibrated one by one to eliminate or minimize nonlinearity and zero drift. This paper presents a systematic design for the testing and calibration process of MEMS-based absolute pressure sensors. Firstly, a numerical analysis is carried out using finite element method (FEM) simulation, which verifies the accuracy of the temperature control of the physical calibration system. The simulation results reveal a slight non-uniformity of temperature distribution, which is then taken into consideration in the calibration algorithm. Secondly, deploying a home-made calibration system, the MEMS pressure sensors are tested automatically and rapidly. The experimental results show that each batch, which consists of nine sensors, can be calibrated in 80 min. The linearity and temperature coefficient (TC) of the pressure sensors are reduced from 46.5% full-scale (FS) and −1.35 × 10^−4^ V·K^−1^ to 1.5% FS and −8.8 × 10^−7^ V·K^−1^.

## 1. Introduction

**Figure 1:**
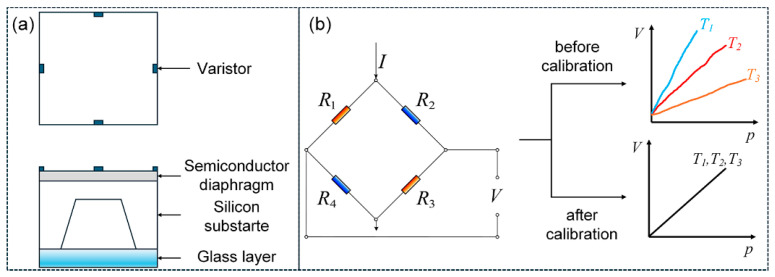
(**a**) The structure and (**b**) electrical output of the absolute pressure sensors.

MEMS pressure sensors exhibit low power consumption, high performance, compact dimensions, mass-production compatibility, and cost-effectiveness [1], and have been widely used in industrial fields [2,3], health examinations [4,5], automotive applications [6,7], and wearable devices [8,9]. Absolute pressure sensors are essential in numerous scenarios requiring precise pressure measurements unaffected by atmospheric pressure changes. Figure 1a shows the core structure of a MEMS-based absolute pressure sensor. It is based on a silicon substrate, one side of which is exposed to the pressure to be measured and the other side faces a vacuum cavity as absolute zero reference. On the surface of the silicon substrate, a Wheatstone bridge circuit composed of four monocrystalline silicon varistors of identical size is manufactured together with a semiconductor diaphragm. On the bottom side of the silicon substrate, the vacuum cavity is sealed with a glass layer. Pressure difference between external pressure and the vacuum cavity causes deformation of the sensing elements, including the diaphragm and the substrate. As a result, the resistance of the varistor varies and the Wheatstone bridge loses its balance. When applying current to the bridge, as shown in Figure 1a, an unbalanced Wheatstone bridge will output an electrical signal related to the pressure [10]. The output signal is greatly affected by temperature, resulting in the problems of zero drift and nonlinearity, which must be compensated or calibrated to a minimum level before application. Figure 1b illustrates the performance of pressure sensors before calibration with dramatical nonlinearity and temperature drift, and the ideal output after calibration.

To overcome these formidable obstacles, engineers have proposed many compensation and calibration methods, which can be mainly divided into two techniques: hardware and software compensation [11]. Hardware compensation refers to the method of adding specific components to the sensor hardware circuit to reduce the temperature drift and zero drift, improving the accuracy of the sensor [12,13,14]. Hardware compensation has fundamental significance for high-temperature pressure sensors and offers good real-time performance, but for general pressure sensors, the accuracy is relatively low and the built-in components are also susceptible to temperature changes [15]. Therefore, with the development of the integrated circuit industry, conditioning chips are gradually being used in the compensation calibration of pressure sensors, and hardware compensation methods are gradually being replaced by software compensation. New methods usually adopt a large amount of experimental data to find out the relationships between variables and realize compensation by precise algorithms. Guo designed a temperature compensation system to modify and compensate temperature drift of the sensor under each temperature point by using the high precision temperature sensor and the self-programed algorithm. It is concluded from the results that the compensated sensor reaches a high accuracy of 0.1% FS in the temperature range of −20 °C to 140 °C and a maximum error of 0.30% FS in the full temperature range (−20 °C to 250 °C) [16]. Ali introduced a polynomial-based highly accurate temperature compensation technique. The measured temperature compensation accuracy is within 0.068% with full scale when temperature varies from 40 °C to 150 °C according to ACE-Q100 [17]. Li proposed a system that integrates temperature measurement and regulation circuits, signal processing, and communication circuits to accurately acquire and transmit pressure sensor data. The experimental results show that the nonlinear error at 50 °C is reduced from the initial 1.82% to 0.24%; the hysteresis error is significantly reduced from 1.23% to 0.046%; and the repeatability error control is reduced from 3.79% to 0.89% [18]. In recent years, with the rise of neural network algorithms, more and more scholars have applied neural network algorithms to the calibration of pressure sensors [19]. Almassri used a novel approach to predict self-calibration in a pressure sensor using a proposed Levenberg Marquardt Back Propagation Artificial Neural Network (LMBP-ANN) model [20]. Wang proposed a temperature compensation method based on an improved cuckoo search optimizing a BP neural network for a multi-channel pressure scanner. The maximum full-scale error of all 32 channels is 0.02% full-scale (FS) error and below in the −40 °C to 60 °C temperature range [21]. Ge used a genetic algorithm-based wavelet neural network to compensate for temperature drifts in the optic fiber FP pressure sensors. Experimental results show that the sensor exhibits high linearity and a sensitivity of 79.956 nm/kPa when the pressure ranges from 0 to 0.1 MPa [22]. The use of neural network algorithms for calibration has high accuracy, but requires high-level hardware performance and massive data samples for training, which is time-consuming when calibrating massive pressure sensors with only slight material and process deviations [23]. The difference between pressure sensors makes it difficult for hardware compensation methods to reach high accuracy, and built-in analog circuits greatly reduce design flexibility and modifiability. Several software compensation methods require extensive test data for parameter fitting, which significantly compromises calibration efficiency. The high computational complexity requires the ANN method to rely on better hardware devices, which greatly increases its cost. Due to the need for a large number of data samples to train the model, the efficiency of the ANN method is relatively low and its latency is high.

To solve the problems mentioned above, this paper presents an automatic and accurate calibration system for absolute pressure sensors. It consists of a pressure chamber with two heating plates in it, a pressure pump, an embedded electronic system, and an upper computer. After calibration, the linearity of the pressure sensor decreased from 46.5% to 1.5%, which is relatively high in accuracy among similar calibration systems. The existing calibration systems typically assume all pressure sensors in a batch maintain identical temperatures at the prescribed setpoint during calibration. However, this conventional approach fails to account for subtle inter-sensor temperature variations, which can compromise calibration accuracy—particularly in systems with inadequate temperature control precision [24,25]. In the system designed in this article, the accuracy of temperature control is well designed and verified by finite element simulation (FEM). A table-driven non-uniform spline interpolation method is used to eliminate temperature differences among the sensor array to be tested. In addition, a novel calibration algorithm, which performs second-order compensation for sensitivity and zero drift, and third-order for output nonlinearity is proposed. The algorithm requires fewer parameters and therefore is time-saving to conduct in a complete test cycle, which takes less than one and a half hours.

## 2. System Design

**Figure 2:**
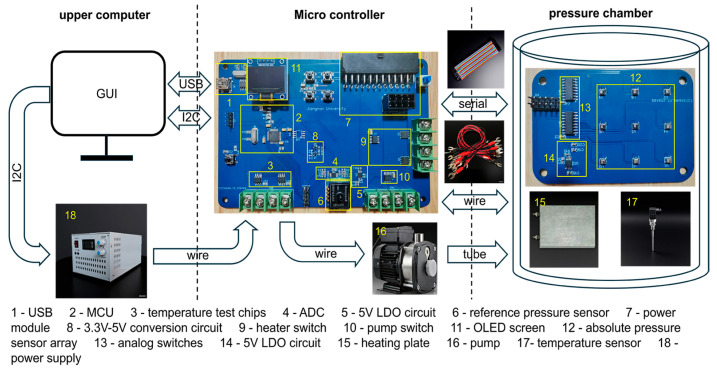
Overview of the pressure sensor calibration system.

Conditioning of the pressure sensors mainly includes two steps, measuring and calibrating, which can be performed within one platform. Figure 2 shows the overview of the sensor calibration system. The platform consists of the following necessary parts:

(1) A PC with measuring and calibrating program;
(2) Programmable power supply;
(3) Vacuum pump;
(4) Embedded system module for temperature and pressure monitoring and control;
(5) Pressure chamber;
(6) Heaters.

Among these parts, the upper computer program and the embedded system module which includes two printed circuit boards (PCBs) are self-developed. One PCB mainly consists of an MCU (STM32F1 from STMicroelectronics, Geneva, Switzerland), a high-resolution analog to digital converter (ADC, ADS1115IDGSR chip from Texas Instruments, Dallas, TX, USA), a 5V LDO circuit (REF5050IDGKR chip from Texas Instruments, Dallas, TX, USA), a pressure reference sensor (ASDXRRX015PDAA5 chip from Honeywell, Charlotte, NC, USA), two temperature sensors (MAX6675ISA+T chip from Analog Devices, Inc., Wilmington, MA, USA), and a communication interface circuit. The power supply can provide all the levels the embedded system needs, such as 3.3 V, 5 V, and 12 V. However, the voltage ripple may not meet the requirement of the ADC, thus reference voltage is needed. Similarly, the reference pressure and temperature sensors are used to determine whether the actual pressure in the chamber reaches the setting values. The heaters and vacuum pump are controlled by the embedded system and eventually follow the upper computer program to set the equipment under test (EUT) to certain temperature and pressure. The communication module uses serial ports to transport information and control signal among the embedded system, the EUT and the upper computer. The secondary PCB incorporates an EUT array, a 5 V LDO circuit, and dual analog switches (74HC4051D, 653 chip from Nexperia, Nijmegen, The Netherlands). The 5 V LDO circuit is designed to provide EUT with suitable working voltage. The analog switches are used to select the pressure sensor whose output value is to be read. The proposed system features a high degree of integration. In a standard calibration system, temperature and pressure control are handled externally. By contrast, the proposed system incorporates integrated temperature and pressure control, enabling fully automated and intelligent calibration across the entire temperature and pressure range via upper computer control.

The default calibration process is as follows:

(1) EUT is fixed in the pressure chamber;
(2) The program in the upper computer sends measurement series to the embedded system;
(3) The embedded system generates the control signals for temperature and pressure control;
(4) The embedded system acquires the measured data and sends it back to the upper computer;
(5) The upper computer program calculates the calibration coefficient according to the measured data.

**Figure 3:**
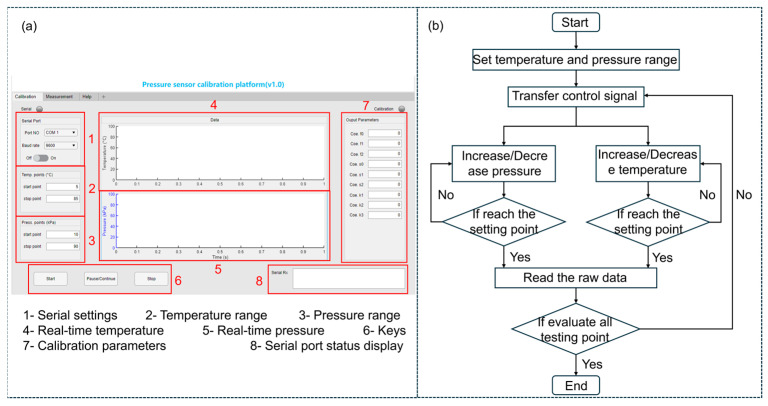
(**a**) GUI design and (**b**) operation process of the program.

In the upper computer, MATLAB is used to design the graphical user interface (GUI) for the calibration process control and calculating calibration parameters. Figure 3a shows the GUI, and Figure 3b illustrates the operation process of the program. After setting the range of temperature and pressure, the program monitors the pressure and temperature of the EUT in real time and displays them on the software interface. When the temperature and pressure reach the set values, the software will read the output voltage of the EUT through the serial port as raw data, calculate the calibration parameters and display them on the interface.

## 3. Simulation Analysis for Thermal Control

### 3.1. Simulation Settings

**Figure 4:**
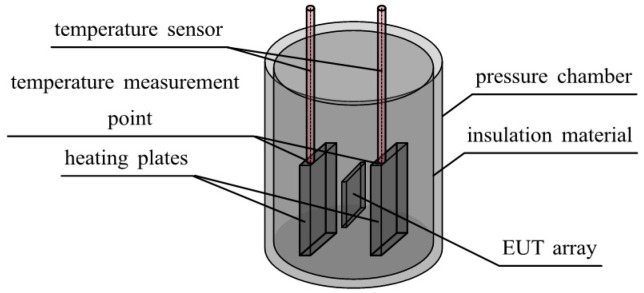
Schematic diagram of three-dimensional structure of pressure chamber.

Temperature exerts a pronounced influence on pressure sensor output, necessitating calibration as a primary mitigation strategy. Therefore, this study conducted thermal simulation of the calibration system to develop precise temperature control and testing methods. The simulation area consists of the pressure chamber, heating plates, insulation layer, and EUT. Figure 4 shows the schematic of the three-dimensional pressure chamber. Finite element simulations were conducted using COMSOL Multiphysics 5.6. Two design schemes (single heating plate vs. double heating plates) were compared, and an optimized temperature control method was determined by analyzing testing temperature and EUT temperature errors. Parameters used in the simulation are listed in Table 1.

#### 3.1.1. Heat Source

Copper heating plates can be approximated as heating sheets with uniform heating power, and their power density can be expressed as:

(1) $$P_{e}=\frac{P_{0}}{V_{0}}$$

where *P*_0_ is the total power of the heat source and *V*_0_ is the volume of the heat source.

#### 3.1.2. Conduction and Convection

(1) The temperature distribution of the air inside the pressure chamber can be described by the following heat transfer equation:

(2) $$\rho C_{p}u\cdot \nabla T+\nabla \cdot (-k\cdot \nabla T)=Q$$

where *ρ* is the density of the fluid, *C_p_* is the heat capacity of the fluid, and **u** is the flow velocity of the fluid. The first term on the left side of the equation can be used to describe the process of heat conduction in the air. The second item on the left can be used to describe the process of heat transfer between the heat source and air, as well as between air and the pressure chamber.

(2) The heat transfer process in the pressure chamber can be described by Fourier’s law:

(3) q=−k⋅∇T

where *k* is the thermal conductivity of the material. The negative sign represents that the heat flux is opposite to the temperature gradient.

(3) The heat transfer process between the pressure chamber and the external environment can be described by the convective heat transfer equation, as follows:

(4) $$q_{0}=h(T_{ext}-T)$$

where ***q*_0_** is the heat flux, *h* is the heat transfer coefficient, *T_ext_* is the external temperature, and *T* is the object temperature. 

For the heat transfer process from the top surface of the pressure chamber to the outside, the following formula can be used to solve the heat transfer coefficient [26]:

(5) $$h=\left\{\begin{array}{l} \begin{array}{cc} \frac{k_{air}}{L}0.54Ra_{L}^{1/4} & T>T_{ext}\;\mathrm{and}\;10^{4}\leq Ra_{L}\leq 10^{7} \end{array}\\ \begin{array}{cc} \frac{k_{air}}{L}0.15Ra_{L}^{1/3} & T>T_{ext}\;\mathrm{and}\;10^{7}\leq Ra_{L}\leq 10^{11} \end{array}\\ \begin{array}{cc} \frac{k_{air}}{L}0.27Ra_{L}^{1/4} & T\leq T_{ext}\;\mathrm{and}\;10^{5}\leq Ra_{L}\leq 10^{10} \end{array} \end{array}\right.$$

where *L* is the characteristic length with a value of 0.02725 m, and *Ra_L_* is the Rayleigh dimensionless number.

For the heat transfer process from the side of the pressure chamber to the outside, the following formula can be used to solve the heat transfer coefficient [27]:

(6) $$h=\left\{\begin{array}{l} \begin{array}{cc} \frac{k}{L}\left\{0.68+\frac{0.67Ra_{L}^{1/4}}{{\left[1+{\left(\frac{0.492k}{\mu C_{p}}\right)}^{9/16}\right]}^{4/9}}\right\} & \begin{array}{cc} Ra_{L}\leq 10^{9} & \end{array} \end{array}\\ \begin{array}{cc} \frac{k}{L}\left\{0.825+\frac{0.387Ra_{L}^{1/6}}{{\left[1+{\left(\frac{0.492k}{\mu C_{p}}\right)}^{9/16}\right]}^{8/27}}\right\} & \begin{array}{cc} Ra_{L}\geq 10^{9} & \end{array} \end{array} \end{array}\right.$$

where *μ* is the viscosity of air, *C_p_* is the heat capacity of air, and *L* is vertical wall height with a value of 0.15 m.

For the heat transfer process from the bottom of the pressure chamber to the outside, as the pressure chamber is placed on an insulated tabletop, Fourier’s law can be used to calculate its heat transfer flux as follows:

(7) q=−k⋅∇T

### 3.2. Simulation Results

**Figure 5:**
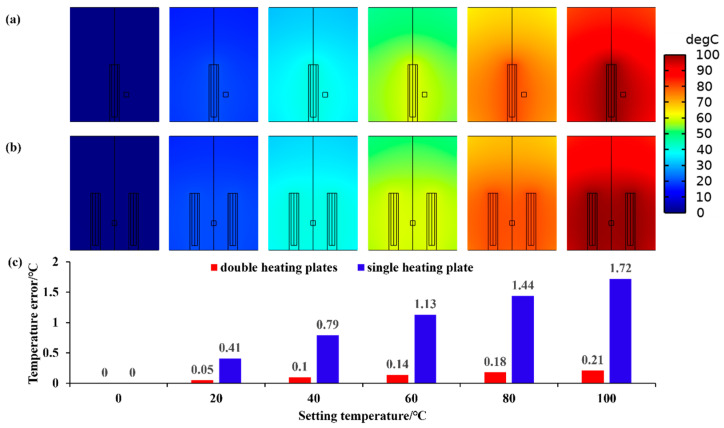
Temperature distribution inside the pressure chamber for (**a**) one single heating plate and (**b**) double heating plate, and (**c**) temperature error between temperature measurement point and EUT.

To calibrate the sensors, six temperature testing points were selected: 0 °C, 20 °C, 40 °C, 60 °C, 80 °C, and 100 °C. Simulations were conducted for each of these six temperature testing points, and a cross-section perpendicular to the heating plates was made through the central axis to reflect the temperature distribution inside the container. Figure 5 shows the temperature distribution inside the pressure chamber and temperature error between temperature measurement point and EUT.

Through simulation, a precise temperature control method can be selected from two aspects: the temperature distribution inside the pressure chamber and the temperature difference between the EUT and the temperature measurement point.

(1) The maximum temperature differences (difference between the maximum and minimum temperature inside the pressure chamber) in the pressure chamber at each temperature testing point are: 0 °C, 4.55 °C, 8.73 °C, 12.59 °C, 16.15 °C, and 19.46 °C by using a single heating plate, and are: 0 °C, 3.79 °C, 7.25 °C, 10.42 °C, 13.35 °C, and 16.27 °C by using two heating plates. From the above data, it can be seen that the extreme temperature difference in the pressure chamber at six different temperature testing points when using two heating plates is smaller than that when using a single heating plate. This reflects that using two heating plates can make the temperature distribution in the pressure chamber more uniform.
(2) The temperature error (temperature difference between EUT and temperature measurement point) is: 0 °C, 0.41 °C, 0.79 °C, 1.13 °C, 1.44 °C, and 1.72 °C by using a single heating plate, and is: 0 °C, 0.05 °C, 0.1 °C, 0.14 °C, 0.18 °C, and 0.21 °C by using two heating plates. By comparing the above data, it can be concluded that the temperature difference between the EUT and the temperature measurement point with two heating plates is smaller than that using a single heating plate. This reflects that using dual heating plates can reduce temperature testing errors.

Comparison of the simulation results indicates that dual heating plates improve temperature uniformity within the pressure chamber and reduce measurement errors. Therefore, two heating plates were used for temperature control of the pressure chamber during the experiment.

## 4. Experiment

As shown in Figure 2, the calibration system primarily comprises a pressure chamber, a microcontroller unit (MCU), and an upper computer constructed by software MATLAB R2019a. The pressure chamber contains the EUT, which comprises nine absolute pressure sensors, a pair of heating plates, a temperature sensor, and a reference pressure sensor.

During operation, the temperature sensor and reference pressure sensor within the pressure chamber transmit real-time temperature and pressure data of the EUT to the MCU, which subsequently relays this information to the upper computer. Concurrently, the micro-controller gets signals from the upper computer and controls the pressure pump and heating element to reach the testing point. The output voltage values of EUT are amplified by the PGA module and stored in the lower computer for raw data acquisition. Once data collection at a given testing point is completed, the upper computer sends temperature and pressure control commands to the MCU via a serial interface, adjusting the chamber’s internal environment to the next testing condition. After all testing points are evaluated, the upper computer retrieves the complete dataset through an I^2^C communication interface. These data are then processed using a calibration algorithm to derive the necessary calibration parameters, ultimately completing the pressure sensor calibration process.

### 4.1. Calibration Algorithm

To reduce the influence of temperature on the output of the pressure sensor, this article proposed a calibration algorithm for pressure sensors, which has low computational complexity, high computational efficiency, and high calibration accuracy. It can compensate for the sensitivity, temperature drift, and zero drift of the pressure sensors.

The calibration algorithm can be represented by the following equations:

(8) $$offset=off+t_{c1}\times (T-T_{0})+t_{c2}\times {\left(T-T_{0}\right)}^{2}$$

(9) $$sensitivity=s_{0}+t_{s1}\times (T-T_{0})+t_{s2}\times {\left(T-T_{0}\right)}^{2}$$

(10) $$V_{out}^{\prime }=(V_{in}-offset)\times sensitivity$$

(11) $$V_{out}=k\times (V_{out}^{\prime }-V_{0})+k_{s}\times {\left(V_{out}^{\prime }-V_{0}\right)}^{2}+k_{ss}\times {\left(V_{out}^{\prime }-V_{0}\right)}^{3}$$

where *offset* represents zero drift and *T*_0_ represents the temperature reference point, which can be taken as the middle value of the total temperature range. Observing the above equation, it can be found that both *offset* and *sensitivity* are related to temperature, which reflects the influence of temperature on the output characteristics of pressure sensors. *V_in_* is uncalibrated voltage, *V*_0_ represents that the calibrated outputs of the pressure sensors across the zero point, and *V_out_* is the final output voltage of the calibrated pressure sensor; *off*, *t_c_*_1_, *t_c_*_2_, *s_0_*, *t_s_*_1_, *t_s_*_2_, *k*, *k_s_* and *k_ss_* are the nine parameters for calibration.

While the proposed system calibrates nine pressure sensors simultaneously, the limited number of temperature measurement points (two symmetric locations) may introduce minor discrepancies between actual and measured temperatures for individual sensors. These discrepancies are related to the location of each pressure sensor. To make the calibration results more accurate, this paper introduces the following formula to describe the influence of the measured temperature and the location of the pressure sensor on the actual temperature of each pressure sensor:

(12) $$T=f(T_{mea},y,z)$$

where *T* is the actual temperature of the pressure sensor, *T_mea_* are measured temperatures, and *y* and *z* reflect the position of each pressure sensor.

**Figure 6:**
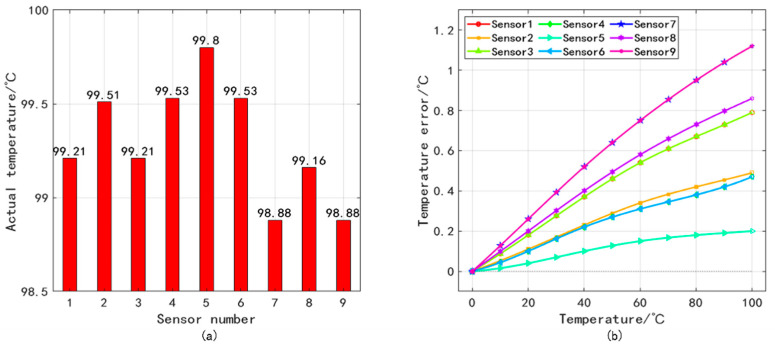
(**a**) Actual temperature of each pressure sensor at 100 °C and (**b**) temperature error of each pressure sensor at different temperature.

Due to the non-linear variation of temperature with the coordinates of the pressure sensor [28], this paper proposes a table-driven non-uniform spline interpolation method to obtain the actual temperature of each pressure sensor. This method combines the lookup table method with cubic spline interpolation, but it can solve the problem of low accuracy of the lookup table method. It requires extracting heat transfer simulation data from nine pressure sensor areas. Due to small number of grid points used in COMSOL Multiphysics for solving, the calculation accuracy cannot meet the required accuracy. Therefore, MATLAB software needs to be used to perform cubic spline interpolation on the data points around each sensor to obtain its actual temperature and temperature error, as shown in Figure 6.

### 4.2. Experimental Results and Analysis

**Figure 7:**
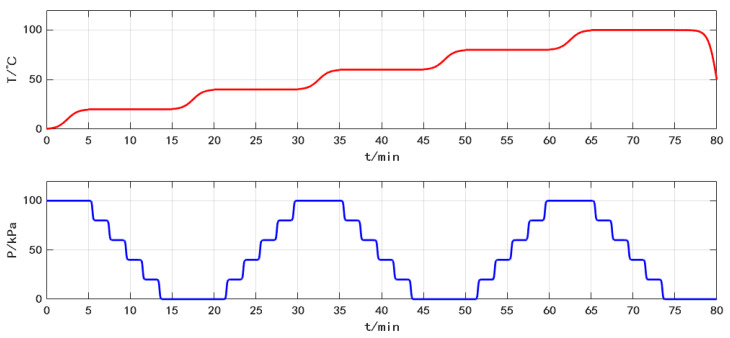
Change of temperature and pressure in pressure chamber.

To improve the pressure sensors’ accuracy, a dynamic calibration method is adopted in the experiment, during which pressure and temperature vary over time. The output voltages of the pressure sensors are measured within a pressure range of 0 to 80 kPa, with a 20 kPa step within a temperature range of 0 to 100 °C with a 20 °C step. The temperature and pressure inside the pressure chamber during the experiment are shown in Figure 7. According to the experimental procedure, after the temperature in the pressure chamber reaches the set temperature, the pressure pump will pump out or fill in air. When the pressure reaches the set value, the MCU will read the output voltage values of the EUT as the raw data. The measurement of a batch of pressure sensors takes about 80 min, which reflects the high efficiency of the calibration system.

**Figure 8:**
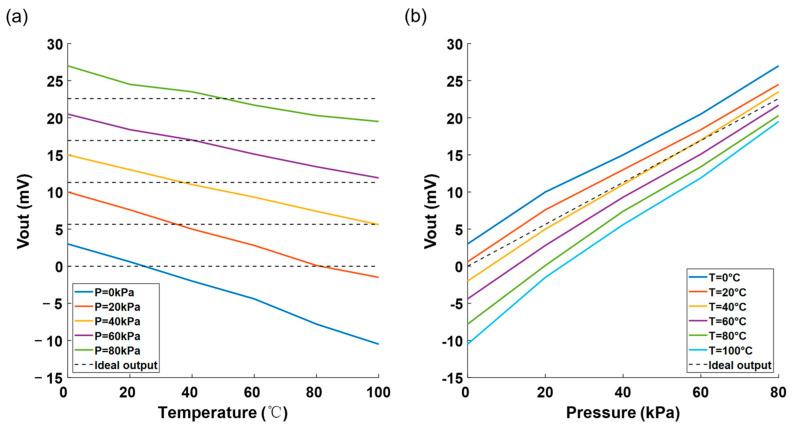
(**a**) Voltage vs. temperature before calibration and (**b**) voltage vs. pressure before calibration.

Figure 8 shows the uncalibrated output voltage against temperature and pressure curves of the pressure sensors. The dashed line in the figure represents the ideal output voltage value of the pressure sensor that isn’t affected by temperature. As shown in Figure 8a, without calibration, the output voltage will vary with temperature (temperature drift). From Figure 8b, it is obvious that the output voltage varies nonlinearly with pressure and has zero drift.

Raw experimental data was brought into the calibration equation to solve the calibration coefficients and obtain the optimal solution of the calibration coefficients by using the nonlinear least squares method. As shown in Table 2, considering the subtle temperature differences between each sensor, nine sets of calibration parameters were obtained. These calibration parameters are relatively similar because the pressure sensors used in this experiment are products from the same wafer. The use of corresponding calibration parameters for different pressure sensors can greatly improve the accuracy of calibration, which is also the key feature of this system.

**Figure 9:**
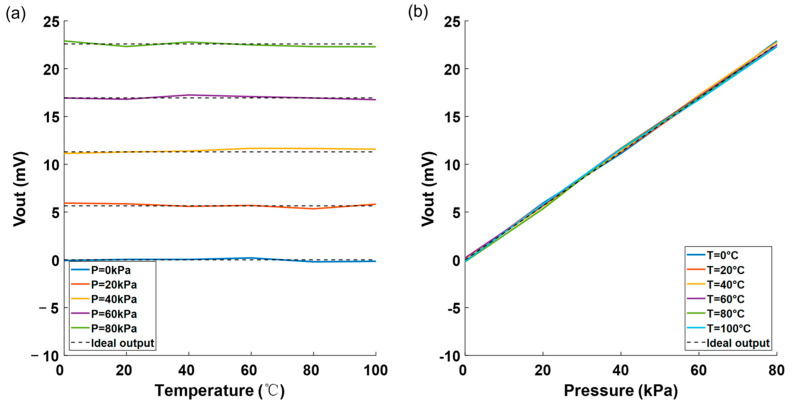
(**a**) Voltage vs. temperature after calibration, (**b**) voltage vs. pressure after calibration.

Figure 9 shows the image of the calibrated output voltage plotted against temperature and pressure using MATLAB. As shown in Figure 9a, under the same pressure, the output voltages of the calibrated pressure sensor exhibit no dependence on temperature. Figure 9b illustrates that the pressure sensor’s output voltages demonstrate good linearity and virtually no zero-offset drift after calibration.

The linearity of a pressure sensor is an important indicator for measuring the degree of deviation between the pressure sensor’s output characteristics and the ideal linear relationship. It represents the percentage of the maximum deviation between the actual output characteristic curve and the theoretical fitted curve within the full measurement range of the sensor to the full-scale output value. Before calibration, the maximum error of the output voltage value is 10.5 mV, and the linearity of the pressure sensor is 46.5% FS. After calibration, the maximum error of the output voltage is 0.339 mV, and the linearity of the pressure sensor is 1.5% FS. The temperature coefficient is reduced from −1.35 × 10^−4^ V·K^−1^ to −8.8 × 10^−7^ V·K^−1^. Table 3 benchmarks this work with other studies. This work achieves high output accuracy, significantly mitigating temperature-induced errors and meeting design specifications.

## 5. Conclusions

This study presents a systematic design for a MEMS-based pressure sensor calibration system. Compared with other systems, a key innovation is the table-driven non-uniform spline interpolation method, which is proposed to minimize temperature testing error among all the sensors in the EUT and FEM simulation, which is used to verify the accuracy of temperature control and distribution. The fabricated circuitry is simple, low-cost, and suitable for small-batch calibration. The system can be applied to the calibration of sensors in a wide temperature range. The results validate that polynomial fitting of empirical data fulfills all sensor conditioning objectives. The linearity and zero temperature coefficient of the pressure sensors are reduced from 46.5% FS and −1.35 × 10^−4^ V·K^−1^ to 1.5% FS and −8.8 × 10^−7^ V·K^−1^. In addition, the calibration system also shows an advantage of fully automation, which utilizes temperature-rise and pressure-drop routines and guarantees the whole operation process takes less than 80 min.

## Figures and Tables

**Table 1:** Parameters used in the FEM model.

Parameter	Value
*T*_0_	273.15 K
*k_air_*	0.026 W·m^−1^·K^−1^
*R_s_*	287.05 J·kg^−1^·K^−1^
*C_p,air_*	1005 J·kg^−1^·K^−1^
*k_steel_*	44.5 W·m^−1^·K^−1^
*ρ_steel_*	7850 kg·m^−3^
*C_p,steel_*	475 J·kg^−1^·K^−1^
*k_copper_*	400 W·m^−1^·K^−1^
*ρ_copper_*	8960 kg·m^−3^
*C_p,copper_*	385 J·kg^−1^·K^−1^
*k_sponge_*	0.08 W·m^−1^·K^−1^
*ρ_sponge_*	60 kg·m^−3^
*C_p,sponge_*	4 J·kg^−1^·K^−1^
*k_silicon_*	130 W·m^−1^·K^−1^
*ρ_silicon_*	2329 kg·m^−3^
*C_p,silicon_*	700 J·kg^−1^·K^−1^
ε	0.6
σ	5.67 × 10^−8^ W·m^−2^·K^−4^
*ρ*	1.29 kg·m^−3^
*V_0_*	0.000036 m^3^

**Table 2:** Calculated calibration coefficients.

	*off*	*t*_*c*1_	*t*_*c*2_	*s*_0_	*t*_*s*1_	*t*_*s*2_	*k*	*k*_*s*_	*k*_*ss*_
1	−2.0706	−0.1316	0	0.7501	−0.0017	0	0.8583	0.0452	0.0015
2	−2.1023	−0.1236	0	0.7500	−0.0010	0	0.8475	0.0414	−0.0011
3	−2.0245	−0.1209	0	0.7324	0.0011	0	0.8356	0.0400	0.0005
4	−2.0923	−0.1024	0	0.7421	0.0003	0	0.8876	0.0345	0.0002
5	−2.1124	−0.1199	0	0.7642	−0.0005	0	0.8723	0.0377	0
6	−2.0894	−0.1342	0	0.7749	−0.0015	0	0.8766	0.0675	0
7	−2.0907	−0.1095	0	0.7295	0.0001	0	0.8459	0.0547	0.0011
8	−2.1010	−0.1456	0	0.7468	−0.0012	0	0.8378	0.0320	−0.0010
9	−2.0698	−0.1506	0	0.7197	0.0009	0	0.8897	0.0521	−0.0007

**Table 3:** Comparison of different calibration methods.

	This Study	Study 1 [13]	Study 2 [16]	Study 3 [21]
Linearity	1.5% FS	2% FS	0.3% FS	0.02% FS
Data samples	Little	Little	Medium	Massive
Latency	Low	Low	Low	High
Integration level	High	High	Low	Low
Flexibility	High	Low	High	High

## Data Availability

The original contributions presented in this study are included in the article. Further inquiries can be directed to the corresponding author.
